# Possible Correlation between Cholinergic System Alterations and Neuro/Inflammation in Multiple Sclerosis

**DOI:** 10.3390/biomedicines8060153

**Published:** 2020-06-08

**Authors:** Valentina Gatta, Guadalupe Mengod, Marcella Reale, Ada Maria Tata

**Affiliations:** 1 Department of Psychological, Health and Territorial Sciences, School of Medicine and Health Sciences, “G. d’Annunzio” University, 66100 Chieti, Italy; valentina.gatta@unich.it; 2 IIBB-CSIC, IDIBAPS, CIBERNED, 08036 Barcelona, Spain; guadalupe.mengod@iibb.csic.es; 3 Department of Medical, Oral and Biotechnological Science, University “G. d’Annunzio” Chieti-Pescara, 66100 Chieti, Italy; m.reale@unich.it; 4 Department of Biology and Biotechnologies C. Darwin, “Sapienza” University of Rome, 00185 Rome, Italy; 5 Research Center of Neurobiology Daniel Bovet, “Sapienza” University of Rome, 00185 Rome, Italy

**Keywords:** cholinergic system, acetylcholine, multiple sclerosis, inflammation, cytokines, EAE

## Abstract

Multiple sclerosis (MS) is an autoimmune and demyelinating disease of the central nervous system. Although the etiology of MS is still unknown, both genetic and environmental factors contribute to the pathogenesis of the disease. Acetylcholine participates in the modulation of central and peripheral inflammation. The cells of the immune system, as well as microglia, astrocytes and oligodendrocytes express cholinergic markers and receptors of muscarinic and nicotinic type. The role played by acetylcholine in MS has been recently investigated. In the present review, we summarize the evidence indicating the cholinergic dysfunction in serum and cerebrospinal fluid of relapsing–remitting (RR)-MS patients and in the brains of the MS animal model experimental autoimmune encephalomyelitis (EAE). The correlation between the increased activity of the cholinergic hydrolyzing enzymes acetylcholinesterase and butyrylcholinesterase, the reduced levels of acetylcholine and the increase of pro-inflammatory cytokines production were recently described in immune cells of MS patients. Moreover, the genetic polymorphisms for both hydrolyzing enzymes and the possible correlation with the altered levels of their enzymatic activity have been also reported. Finally, the changes in cholinergic markers expression in the central nervous system of EAE mice in peak and chronic phases suggest the involvement of the acetylcholine also in neuro-inflammatory processes.

## 1. Acetylcholine and Immune System

Acetylcholine (ACh) is known as one of the main neurotransmitters in the central (CNS) and the peripheral nervous system (PNS) [1]. It plays relevant roles in several organisms: in multicellular organisms and in particular in mammalian species, it mediates the communication between the nervous system and several peripheral organs such as muscle cells, heart, glands, gut, etc…..[2,3]. Interestingly, ACh can be produced also by non-neuronal cells, such as keratinocytes and endothelial cells, contributing to local regulation of the cell physiology [4,5,6].

Evidence has been accumulated over the past 20 years on the ability of immune cells to respond to cholinergic stimuli, focusing on the cholinergic modulation of the immune response and inflammatory processes both in the immune system and in the brain [7,8].

The first evidence suggested that the vagus nerve was responsible of the control of immune cells responses by inflammatory reflex mediated by the cholinergic stimulation [9]. Albeit the vagus nerve stimulation can modulate the peripheral inflammation, it has been proposed that it may control ACh production directly by the immune cells in the spleen or in other lymphoid organs through adrenergic stimulation [10,11]. This hypothesis appears more realistic considering that the ACh has an extremely short half-life in vivo as a consequence of the ubiquitous distribution of its hydrolytic enzymes acetylcholinesterase (AChE) and butirylcholinesterase (BChE) [12], which rapidly degrade it. Therefore, the cholinergic source of ACh must be very close to the site of action (paracrine function). It has been shown that several immune cells are able to produce ACh and to respond to cholinergic stimuli. T and B cells, macrophages and dendritic cells (DCs) express most of the components of the cholinergic system [13,14]. The presence of choline acetyltransferase (ChAT) and AChE in immune cells has been demonstrated by different techniques [8,15,16]. Immunological activation of T cells up-regulates cholinergic activity, as well as the toll-like receptor activation by selective agonists, induces ChAT expression in DCs and macrophages [7]. These data support a clear cholinergic involvement in the regulation of the immune functions.

The acetylcholine signaling in the immune system is mediated by two types of cholinergic receptors: the muscarinic and nicotinic receptors.

The muscarinic receptors (mAChRs) are metabotropic receptors able to activate different signal transduction pathways [17]. The immune cells express muscarinic receptors whose selective activation modulates the synthesis and production of pro-inflammatory cytokines contributing to modulating the inflammatory processes and, albeit they are not involved in the regulation of the antibodies’ synthesis, they favor the antibody class switching from IgM to IgG1 [18,19].

Nicotinic receptors (nAChRs) are classical ionotropic receptors. The immune cells express different nicotinic receptor subunits (α2, α4, α 5, α9, α7 and β1, β2) whose combination contributes to the hetero- or homopentameric receptor subtype formation. The expression of these receptors is differently distributed in the immune cells and in some cases correlates with their differentiation state [20]. The homopentameric α7 nAChR is the best characterized nicotinic receptor subtype in the immune system. This receptor appears mainly involved in the inhibition of inflammatory processes modulating the production of the anti-inflammatory cytokine, suppressing dendritic cells and macrophage activity and leading to the suppression of T cell differentiation [21,22].

Considering the relevant role of the ACh and its receptors in immune cells functions and in the modulation of the inflammatory processes, it is not difficult to hypothesize that a better understanding of the roles played by ACh and its receptors in inflammatory-related diseases such as neurodegenerative diseases, is of great clinical relevance.

In the present review, we summarize the data indicating the possible involvement of ACh also in demyelinating disease such as multiple sclerosis (MS), reporting recent data demonstrating how the alteration of the cholinergic system activity is present in MS patients and in experimental autoimmune encephalomyelitis (EAE) mice, may be correlated with the immune system dysfunction and alterations of the neuro-inflammatory processes characterizing the MS pathology.

## 2. Cholinergic Dysfunction in Multiple Sclerosis

MS is an autoimmune disease characterized by demyelination and chronic inflammation. The progressive phases of the disease are associated with myelin sheets alteration and degradation and decreased oligodendrocyte differentiation. The involvement of the cholinergic system in MS is poorly known. However, several studies on EAE mice have suggested how the treatment with cholinesterase inhibitors may improve motor and cognitive impairment and reduce the neuro-inflammation [23]. These effects were significantly abolished by α7 nAChR antagonists [24]. Moreover, as reviewed by Nizri and Brenner [25], the α7 nAChRs also play a relevant role in the immune system where they control the number of dendritic cells and the proliferation of the autoreactive T cells [26].

According to this evidence, the ACh production and cholinergic marker expression and activity in serum and cerebrospinal fluid (CSF) of MS patients were reported [27]. The first data have demonstrated that ACh levels were significantly lower in MS patients with a relapsing–remitting course of the disease (RR-MS) compared with healthy subjects (HS), both in sera and CSF [27,28]. However, no relationship was found between ACh levels and patient gender or demographic and clinical aspects [27].

Interestingly the enzymatic activity of the ACh hydrolyzing enzymes AChE and BChE resulted significantly increased in sera of MS patients [29]. The studies of the expression of AChE and BChE transcripts in peripheral blood mononuclear cells (PBMC) of MS patients have demonstrated an increase in the transcript levels of both hydrolyzing enzymes, in particular for BChE [28,29]. Moreover, the levels of ACh biosynthetic enzyme ChAT were higher in MS patients compared with HS. Finally, the expression of transcript for OCTN-1 and mediatophore, the two proteins typically expressed in immune cells and responsible for the non-vesicular ACh release were also upregulated in MS patients [29].

**Figure 1:**
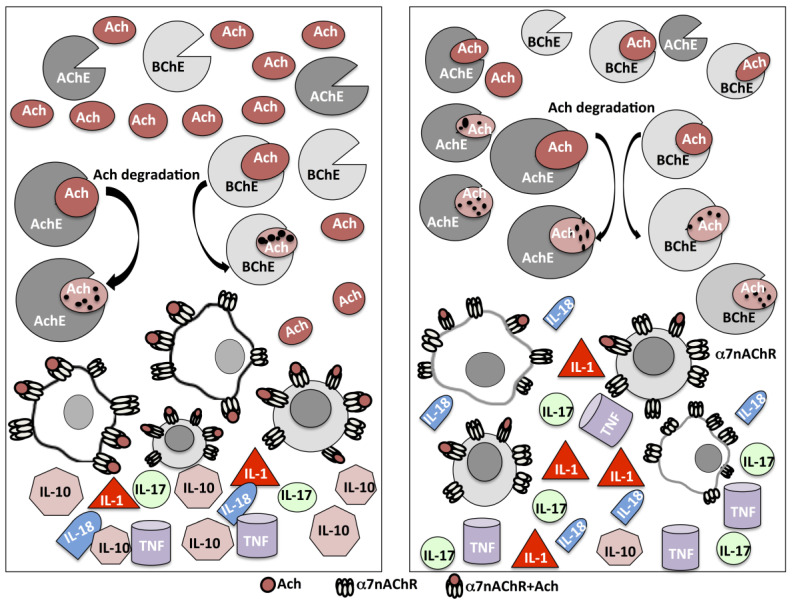
Schematic representation of the mechanism associated with Acetylcholine (Ach) degradation by acetylcholinesterase (AChE) and butirylcholinesterase (BChE) (see arrows), in healthy (left) and multiple sclerosis patients’ immune cells (right). The decreased ACh levels may contribute to a reduced α-7 nicotinic receptor activation and a consequent increase of pro-inflammatory cytokines.

These results suggest that the decreased levels of ACh observed in MS patients, may mainly be dependent on the increased expression and activity of cholinergic hydrolyzing enzyme BChE and AChE (Figure 1).

The dysregulated homeostasis of ACh has already been described in other neurological and neurodegenerative diseases (i.e., schizophrenia, Alzheimer’s, Parkinson diseases) [30]. For these pathologies, as well as for MS, it is difficult to understand whether the cholinergic dysregulation is the cause or the effect of the pathology.

Interestingly, also some autoimmune diseases present alterations of cholinergic receptors expression or activity (i.e., Sjogren’s disease, sepsis, myasthenia gravis) [31,32,33], confirming a relevant role of cholinergic receptors also in immune system activity. Thus, the MS may be a new pathology possibly correlated with cholinergic dysfunction.

## 3. The Interface between Cholinergic System and Inflammatory Cytokines in MS

Cytokines orchestrate all phases of immune responses, act in highly complex and dynamic networks in a paracrine and/or autocrine manner and often perform overlapping and partly redundant functions through multicomponent molecules that can be shared by different cell types. For the homeostatic balance, a dynamic equilibrium between pro and anti-inflammatory cytokines is required. Cytokines play a key role in the pathogenesis of MS through the activation of the immune system in the periphery and in the central nervous system. Aberrant immune responses occur in MS and it is likely that the spectrum of the cytokines produced decisively influences the outcome of the disease, as evidenced by the cytokine profiles altered in the central nervous system [34] and in the biological fluid of MS patients. As reported above, the serum levels of ACh were found to be lower in MS patients with respect to levels detected in HS. Considering that ACh plays a central role in the crosstalk between the immune and nervous system and that may act as a suppressor of inflammatory responses, the relationship between the ACh synthesizing and degrading enzymes and pro-inflammatory cytokines was evaluated. Interestingly, AChE and BChE are present at high levels in MS patients, contributing to alter the steady-state equilibrium of the ACh [29]. The balance between the levels of pro- and anti-inflammatory cytokines and their sequential release can be decisive for the severity of inflammatory responses. Alteration of this balance can convert a beneficial effect into a pathological inflammatory reaction. The well-known contribution of the inflammatory component to the progression of multiple sclerosis has led to new attempts to discover ways to attenuate inflammation. The cholinergic system has been suggested as a mediator of the crosstalk between the immune and nervous systems playing an important role as a modulator of immune responses. The effect of dysregulated balance between ACh, AChE and BChE on cytokines levels was studied in order to understand the role of the cholinergic system on inflammatory environment present in MS patients. In the CSF and sera of RR-MS patients and control subjects, the levels of IL-10 and IL-4 were evaluated and no statistical differences were detected [27]. Conversely in both CSF and serum from RR-MS patients, the levels of IL-1β were significantly higher than those detected in CSF and serum of control subjects. Interesting, in both MS and control groups, also the mean level of IL-17 was higher in the CSF than in serum, and serum levels of IL-1β were about four times higher than in CSF of MS subjects. These data confirmed the active pro-inflammatory state characterizing the MS patients [27,28,35]. Moreover, significant differences in TNFα, IL-12/IL-23p40 and IL-18 levels were observed between MS patients and controls. However, a not significant association between ACh hydrolyzing enzymes and the levels of these cytokines was found [27,29].

However, in the serum of MS patients undergoing IFN-β treatment substantially lower levels of IL-1β, IL-12/23p40, IL-18, TNF-α, and HMGB-1 and higher levels of IL-18BP and ACh were detected. These results, other than reinforce the previous observation that a reduction in ACh levels is involved in RR-MS and that ACh and circulating cytokines are mutually influenced [29], have suggested the ability of IFN-β to modulate inflammatory cytokines also by restoring of the ACh levels [36].

ACh was present in inflammatory sites, probably synthesized from immune cells, and cytokines produced to represent an inflammatory input signal to activate afferent vagus nerve fibers that transmit information to the brain. Therefore, the cytokines produced by blood peripheral cells might affect the function of the CNS by crossing the blood–brain barrier (BBB) for direct interaction with CNS tissue. On the other hand, the cells of the immune systems may respond to neurotransmitters released by autonomic nerves and communicate through neurotransmitters [11].

The higher gene expression level of several cytokines, such as IL-1β, IL-12/IL-23p40, IL-17, IL-18 and TNFα was detected in RR-MS compared to HS, according to with the levels measured in the serum; this confirms the relationship between expression and production of pro-inflammatory cytokines in MS patients [35].

These results led to evaluating which components of the cholinergic system could be relevant in terms of the therapeutic tool in controlling the inflammatory state in MS patients. It is known that α7 nicotinic receptors stimulate the cholinergic anti-inflammatory pathway [37]. The expression of α7 and α4 nicotinic receptor subunits, in RR-MS patients and HS, was documented [35]. The expression of the α7 receptor protein by western blot analysis, confirmed the gene expression data, showing higher protein levels in MS patients. The stimulation with phytohaemagglutinin (PHA) was also able to differently modulate the α7 nicotinic receptor expression in RR-MS patients and HS. In fact, PHA stimulation significantly decreased mRNA levels for the α7 receptor subunit only in RR-MS patients. Instead, the increased α7 nAChR expression after nicotine stimulation in PBMC of RR-MS patients correlated with a reduction of the pro-inflammatory cytokines [35]. In fact, the ability of nicotine to modulate the inflammatory cytokines in RR-MS patients was also evaluated. The effects of nicotine on the production of IL-1β and IL-17 from PHA-stimulated PBMC of RR-MS and HS were described. In both groups, nicotine decreased the levels of IL-1β and IL-17 released, but a more significant decrease was observed in RR-MS patients. The expression of the cytokine transcript levels after nicotine treatment was also evaluated [35]. In particular, the treatment of PBMC with PHA plus nicotine significantly reduced the expression of IL-1β, especially in MS patients. Interestingly the co-treatment of PBMC with PHA and 10 μM nicotine did not modify the expression of α7 subunit mRNA in HS, while inducing a significant increase of α7 transcript levels in RR-MS patients [35]. Globally, these data suggest that the locally expressed and released components of the non-neuronal cholinergic system may play paracrine or autocrine effects and contribute to modulate inflammatory cytokines (Figure 1). This may suggest strategies involving the use of selective agonists of α7 nAChRs in the modulation of the inflammatory responses also in demyelinating disease.

## 4. Genetic Polymorphisms for BChE and AChE and MS

**Figure 2:**
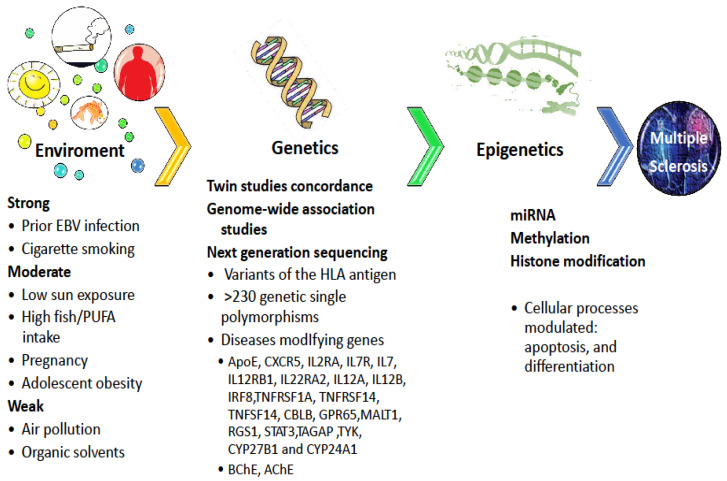
Risk factors for multiple sclerosis (MS). A hereditary basis of MS is suggested by epidemiological studies and recurrence risk of MS in twins, siblings, conjugal MS individuals. MS is believed to result from multiple independent or interacting polymorphism genes as well as their interaction with behavioral and environmental factors with strong, moderate, or weak effects. Epigenetic mechanisms that have been detected for MS pathogenesis are DNA methylation, histone modification and some microRNAs alternations.

The etiology of MS can be considered multifactorial, based on the interaction between genetic and several environmental factors (Figure 2). A hereditary basis of MS is suggested by epidemiological studies. This can be supported by the recurrence risk of MS in twins, siblings, conjugal MS individuals, and lower recurrence risk in adoptees [38,39,40]. In the general population, the risk of developing MS is about 0.00125%, including the variability for geographic areas. Overall, about 15% of the patients with MS have an affected relative. Studies in twins reveal a concordance of 25% in monozygotic twins and only 2.4% in dizygotic twins [41]. Recently, the following meta-analysis estimate was reported: 0.50 (95% CI: 0.39–0.61) for heritability, 0.21 (95% CI: 0.11–0.30) for shared environmental component and 0.29 (95% CI: 0.26–0.33) for unique environmental component [42]. The authors conclude that these results support the efforts to fill the gap of ‘missing heritability’ by genome-wide association studies (GWAS) on large populations as well as massive sequencing analysis by next-generation sequencing (NGS). The risk of developing the disease is 15-fold higher when MS is present in a first-degree relative. Siblings of patients with MS have a risk of ~2.6%, parents have a risk of 1.8% and sons have a risk of ~1.5%. Accordingly, considering the distinction between monogenic and complex traits, no single gene or environmental factor causes MS and it does not follow the Mendelian model.

MS is believed to be dependent on multiple independent or interacting polymorphism genes with small or moderate effects, as well as their interaction with behavioral and environmental factors (Figure 2) [43]. Several studies also aim to disclose the genetic basis of MS. For decades, only several variants of the *HLA* antigen were known to have the strongest effect on the risk of MS [44,45]. Carrying HLA-DRB1*1501 is associated with about three-fold greater odds of developing MS, while carrying HLA-A*02 is associated with meaningfully reduced odds. So far, genome-wide association studies, carried out in populations worldwide, provided strong evidence for the association of approximately >230 genetic variants. All these variants only account for 20% to 30% of MS heritability, suggesting that its remaining part is likely related to epigenetic factors and gene-gene or gene-environment interactions. Considerable attention has been focused on studies evaluating disease-modifying effects in MS that identified genes such as the *APOE*, *CXCR5*, *IL2RA*, *IL7R*, *IL7*, *IL12RB1*, *IL22RA2*, *IL12A*, *IL12B*, *IRF8*, *TNFRSF1A*, *TNFRSF14*, *TNFSF14*, *CBLB*, *GPR65*, *MALT1*, *RGS1*, *RIC3*, *STAT3*, *TAGAP*, *TYK2*, *CYP27B1* and *CYP24A1* [44,45].

Recently, it was reported in genome-wide association studies, that the implication of RIC3, a chaperone of nAChRs, in multiple sclerosis (MS) and neuroinflammatory disease. RIC3 promotes the functional expression of α7 nAChR, and it was shown a dynamic regulation of RIC3 in macrophages and in lymphocytes, following an immune activation in human and murine cells.

Moreover, increased average expression of *RIC3* and *CHRNA7* in lymphocytes from MS patients but not in healthy donors was observed. These data are consistent with a role for RIC3 and in its regulated expression in inflammatory processes and in neuroinflammatory diseases [45].

The variation in some genes of the cholinergic system, such as those encoding BChE (gene *BChE*, chrom. 3q26.1–q26.2) and AChE (gene *AChE*, chrom. 7q22.1) [http://www.genatlas.org] has also been studied in MS patients in relation to altered levels of their enzymatic activity.

Variants in these genes have been largely investigated in relation to the onset of Alzheimer’s disease [46,47], since the levels of these enzymes were found increased in the brains of patients, as well as a number of low-grade systemic inflammation pathologies [48,49].

In particular, two specific polymorphisms within these genes have been studied, namely rs1803274 for *BChE* and rs2571598 for *AChE*. It is reported that rs1803274 for *BChE* is able to reduce the enzymes’ activity by about 30%–50% [50]. The *BChE* rs1803274 is characterized by a G/A substitution inducing the Ala/Tr change at the codon 539 and it produces the so-called K-allele. This variation causes the reduction of 30%–60% of ACh’s hydrolyzing activity and 30% of the capacity to hydrolyze butyryl-thiocholine. *BChE* K-carriers, showing lowered hydrolytic activity of *BChE* K-allele, could potentially improve cholinergic activity.

There are 19 single nucleotide polymorphisms (SNPs) for *AChE* [51]. The only rs1799806 on exon 6, a functional intronic C/T substitution, was associated with activity changes. The AChE activity of homozygote Pro/Pro genotype was significantly lower than Arg/Arg genotypes. The presence of these variants is controversially considered a risk factor for Alzheimer’s disease, as well as other conditions such as stroke, Parkinson’s disease and related dementia [52,53]. Even if cholinergic neurotransmission regulates the immune response and inhibits cytokine release after stroke, only the variant alleles of *BChE* have been identified as risk factors for ischemic stroke [54] and associated with reduced BChE activity in patients with post-stroke dementia (PSD) [49], while *AChE* rs1799806 do not influence the AChE activity.

As above-described the ACh levels in serum of RR-MS patients were inversely correlated with the increased activity of the hydrolyzing enzymes AChE and BChE [29]. The observed lower circulating ACh levels in sera of MS patients are probably dependent on the higher activity of cholinergic hydrolyzing enzymes [29].

In this context, it was recently investigated whether lower ACh concentration observed in RR-MS compared to HS may be related to the ability to regulate the extracellular ACh levels, when needed, or to the variation of AChE or BChE activity related to the association with rs1803274 for *BChE* and rs2571598 for *AChE* genetic variations [55]. An association between the *BChE* K-allele and *AChE* rs2571598 in 102 relapsing remitting-MS patients compared to 117 healthy controls was reported. These data underlined that in RR-MS patients carrying the K-allele, higher BChE activity was observed [55]. Although this is in contrast with the reported role of this SNP in *BChE* gene, this excess of circulating BChE removes ACh, negatively influencing the levels of all investigated pro-inflammatory cytokines (Figure 1). Conceivably, other pathways, such as increased transduction of BChE, microRNAs or other gene modulators acting in combination with BChE-K, could be involved in the balance of the BChE as well as a higher BChE enzymatic activity induced by the presence of the polymorphic allele may reduce the amounts of circulating ACh.

These evidences may suggest that specific genetic endo-phenotypes are able to modulate the pro-inflammatory immune responses in MS patients, altering the activity of AChE through ACh hydrolysis. These data highlight the potential role of the non-neuronal cholinergic system in immune cell function, even if studies on larger populations are needed in order to better discover MS etiology and progression and to develop new disease-modifying therapies for MS with AChE and ACh as targets.

The reported literature about genetic biomarkers in MS is not always consistent in the worldwide population. The detection of a single nucleotide polymorphism related to MS pathogenesis is very hard given that several genetic polymorphisms are implicated, each with a small contribution to the susceptibility or resistance to MS. Although numerous causal genes have been detected by genome-wide association studies (GWAS), these susceptibility genes are linked to relatively low disease risk, indicating the important role of environmental factors in the pathogenesis of the disease. Next-generation sequencing (NGS) technology, with high-throughput capacity and accuracy, could be a powerful tool to discover the genetic basis of MS. NGS can be applied to sequence analysis in any part of the genome and the resulting transcriptome, including the whole genome, exons, and other interesting regions, and accordingly can be roughly classified as whole-genome sequencing (WGS), whole-exome sequencing (WES), RNA sequencing (RNA-seq), and DNA methylation sequencing. So far, only rare variants of modest effect on MS risk affecting a subset of patients have been detected by NGS including *CYP27B1* and *TYK2* genes [56,57]. Epigenetic mechanisms that have been detected for MS pathogenesis are DNA methylation, histone modification and some microRNAs’ alternations. Several cellular processes including apoptosis, differentiation and evolution can be modulated along with epigenetic changes [58]. All these data support the idea of a complex basis of MS with clear gene–environment interactions and epigenetic alterations triggered by environmental exposures in individuals with a particular genetic profile (Figure 2) [59].

## 5. Cholinergic Markers Alteration in the Brain of EAE Mice 

One of the animal models of MS, EAE can be obtained after active immunization with myelin oligodendrocyte glycoprotein (MOG) 35–55 peptide as antigen in C57BL6 mice, and reproduces many pathological features observed in MS [60]. Several days after immunization, it is possible to observe immune cells infiltrating the CNS and activation of microglia. These events take place predominantly in the spinal cord but also in other brain structures such as the cerebellum and the optic tract [61], which lead to axonal damage and demyelination [62]. The MOG_35-55_-induced model of EAE is characterized by the peak inflammatory phase, followed by a chronic phase of neurological deficit.

ACh may play a role in the development or in the remission of MS. Recent analyses of cholinergic markers in the brain and spinal cord of EAE mice revealed specific alterations in their expression in different brain areas and cellular populations associated to the different phases of the disease [63]. The mRNA coding for ChAT, the enzyme responsible for the synthesis of ACh from choline and acetyl-CoA that serves as a marker of cholinergic cells, presents alterations in the expression levels in different brain regions and spinal cord and in different disease phases of EAE mice [63]. Several cholinergic nuclei of EAE mice in the chronic phase present an increment in ChAT expression levels and an increase of the AChE activity and mRNA expression at peak of disease, suggesting that the balance of ACh levels may contribute to the chronicity of the disease.

**Figure 3:**
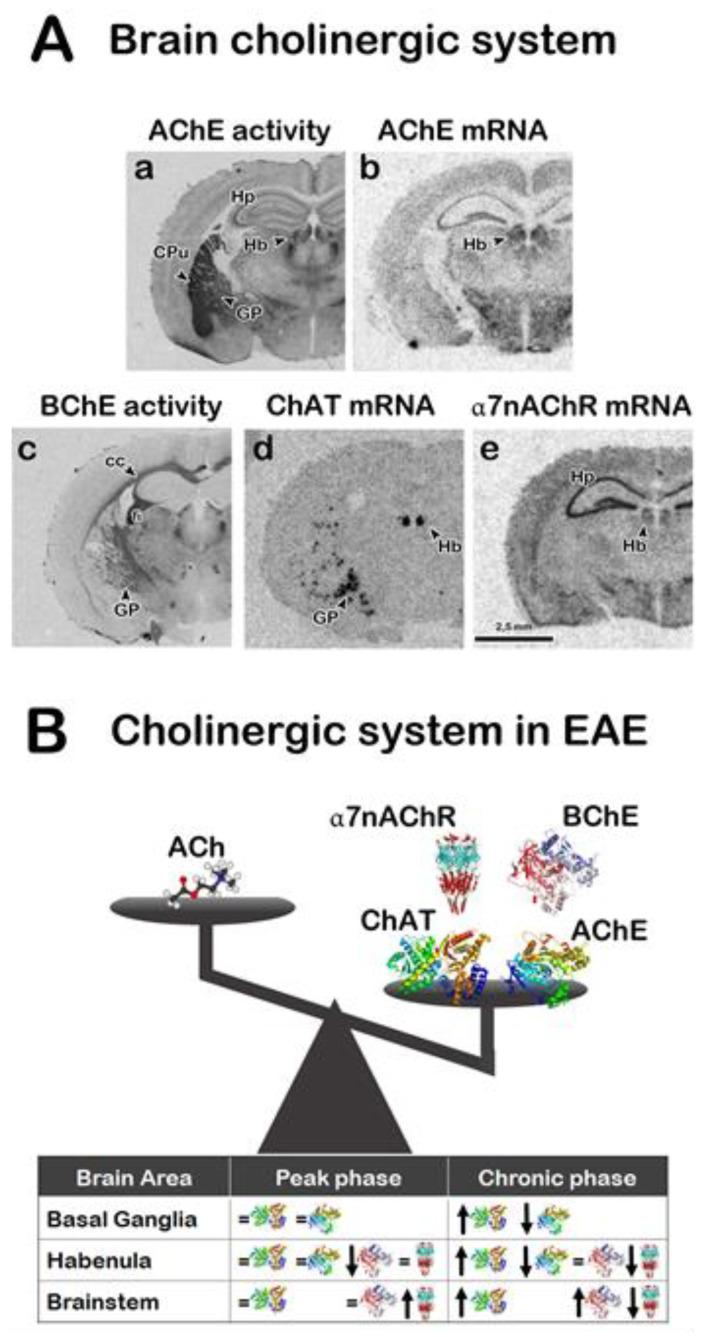
Cholinergic system changes in some brain areas (basal ganglia, habenula, and nuclei of the brainstem) of experimental autoimmune encephalomyelitis (EAE) mice at peak and chronic phases of the disease. (**A**) Photomicrographs showing AChE (**a**) and BChE (**c**) enzymatic activities stained histological brain sections and photomicrographs from autoradiograms of control mouse brain tissue sections visualizing brain areas expressing mRNAs coding for *AChE* (**b**), *ChAT* (**d**) and *α7nAChR* (**e**). (**B**) Representation of the homeostatic balance of cholinergic markers expression in brain areas of EAE mice. The table summarizes the quantification of enzymatic activities and mRNAs expression of cholinergic markers at peak and chronic phases of the disease. Abbreviations and symbols: CPu, caudate-putamen; CC, cerebral cortex; fi, fimbria; GP, globus pallidus; Hb, habenula; Hp, hippocampus; 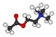, ACh; 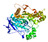, AChE; 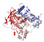, BChE, 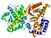 ChAT; 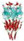, α7nAChR.

The BChE activity, a non-selective cholinesterase enzyme that catalyzes the ACh hydrolysis, is altered in the habenula and solitary nucleus in both disease phases of EAE mice and in higher proportion in astroglia and microglia/macrophage cells in the chronic group [63]. BChE activity is detected in both glial cell populations and presents a significant increment in EAE animals in the chronic phase compared to the peak phase or control brains. The high ratio of BChE to AChE found in astrocytes of EAE mice in peak phase [63] may suggest this ratio as a potential marker for brain areas selectively vulnerable to this neuropathology as it was described for Alzheimer’s disease brains [64]. However, the treatment of EAE mice with a BChE/AChE inhibitor (rivastigmine) resulted in an amelioration of the clinical symptoms associated also with a reduction in the demyelination [24], supporting the role of BChE in neuroinflammation and demyelination (Figure 3).

As reported above, several studies show that α7 nAChR is the key subtype involved in nAChR-mediated immune regulation [20,26,65]. α7 nAChR mRNA expression in the EAE chronic group is increased in habenula and decreased in the dorsal tegmental nucleus. Conversely, the mRNA levels of this receptor are increased in the dorsal tegmental nucleus of animals in the peak phase [63]. The altered expression of this receptor subunit in EAE mice during the chronic phase compared to the peak phase of the disease may sustain its role in the neuro-inflammation regulation also in the brain (Figure 3).

## 6. Cholinergic Receptors in the Glial Cells

New ideas have emerged during the past several years on how the neurotransmitters, including Ach, can actively participate in neuron–glia cross talk. This interaction is relevant both during nervous system development to improve and address neuron and glial cell survival, proliferation and differentiation, and in adulthood, to maintain axonal function and myelin integrity [66]. It is now known that glial cells express receptors for different neurotransmitters, suggesting that these molecules, most probably when released in extra-synaptic regions [67,68,69], may modulate glial cell survival, proliferation, and myelination [67,70].

The expression of ACh receptors has been reported in several glial cells indicating these cells as potential targets for ACh action [70,71]. Oligodendrocyte (OLs), the main target in MS, express muscarinic receptors whose activation triggers different signal transduction pathways [67,70,72,73,74]. The presence of nicotinic receptor subunits (*α*3, *a*4, *a*5, *a*7, *β*2, and β4) was also revealed by RT-PCR and immunocytochemistry in oligodendrocytes precursor cells (OPC), but not in mature cells [75]. Oligodendrocytes express also acetylcholine muscarinic receptors. M1, M3, and M4 ACh receptors were the muscarinic subtypes expressed in OPCs, whereas all five muscarinic receptor subtypes were found to be expressed at low levels in mature OLs; thus muscarinic receptors favor the maintenance of immature proliferating progenitor cells and counteract progression toward a mature state [75]. Accordingly, with this evidence, more recent research demonstrated that antagonists of muscarinic receptors promote oligodendrocyte differentiation and rescue of the lesions in white matter in EAE mice [76]. In fact benztropine, a typical muscarinic antagonist used as a drug for Parkinson’s disease treatment has been proposed for MS treatment considering that MS symptoms in the EAE mouse model were reverted [76,77]. Moreover, the benztropine appears to favor oligodendrocyte differentiation and myelination by a specific antagonism of M1 and M3 receptors on oligodendrocyte precursor cells (OPCs) [76]. Another important aspect to consider is that muscarinic receptors are able to modulate pro-inflammatory cytokine production [18,28], so it may be possible to hypothesize that the antagonism of muscarinic receptors can support not only oligodendrocyte differentiation but also the neuro-inflammation decrease.

The presence of muscarinic receptors was demonstrated in cultured astrocytes [78] as well as the expression of functional nAChRs subunits (*a*4, *a*7, *β*2, *β*3) has been reported in rat astrocytes [39,79]. In particular, the expression of *a*7 nAChR in astrocytes plays a relevant role in neuroprotection in several neurodegenerative diseases (i.e., Alzheimer and Parkinson) [80,81].

Finally, microglia also express both muscarinic and nicotinic receptors. Zhang and collaborators [82] described for the first time the expression of functional muscarinic receptors in human microglia. Interestingly the upregulated expression of M3 receptor subtype in these cells causes an increase of MHC-I and MHC-II expression, suggesting the pro-inflammatory microglia phenotype [83]. Microglia also display nicotinic receptors, with a prevalence of *a*7 subunit [84,85,86,87]. For these reasons, this nicotinic receptor subtype may be considered an interesting therapeutic target for several neurological disorders considering its ability to modulate the cholinergic anti-inflammatory pathway and the synaptic plasticity.

Taking all these into account, it cannot be excluded that cholinergic stimulation in the brain may influence a complex network controlling both neuronal and glial functions and play a relevant role in the control of the neuro-inflammation.

## 7. Conclusions

Understanding the mechanisms leading to aberrant immune response and the severe neuro-inflammation in MS plays a relevant role in the development of new treatments directed to decrease pro-inflammatory cytokine production and to ameliorate the clinical symptoms, slowing the disease outcome and delaying or arresting the onset of the disabilities. In this review we have presented recent data in EAE mice and in MS patients, supporting the role of ACh in MS. The dysregulated ACh homeostasis in MS, suggest how its altered functions might represent an additional pathogenetic mechanism negatively influencing the cytokines production with consequent disease outcome.

Interestingly, using the EAE mice model, it has also been described as the alteration of ACh synthesis or degradation may impact on neuron function, contributing to the motor and cognitive disabilities characteristics of MS patients, also influencing the glia network, the differentiation and survival of the oligodendrocytes, and triggering the neuro-inflammation.

The relevant role played by ACh in this context, is supported by the evidence that modulating the cholinergic system activity or cholinergic receptor functions, the clinical disabilities, at least in mice, are significantly reduced. In fact, the altered balance of ACh also in cholinergic area of the brain or spinal cord of EAE mice (i.e., basal ganglia or spinal motoneuros), suggested that the decreased ACh levels may influence the MS motor disabilities both reducing the levels of the neurotransmitter in the cholinergic neurons and increasing the neuro-inflammation with consequent impact on myelin disruption and axonal conduction [63,76,77].

This aspect appears supported by additional studies performed with conventional drugs used in the treatment of MS, as INFβ or dimethyl fumarate [36,88], that demonstrate a significant amelioration of the cholinergic activity with a possible consequent reduction of the clinical symptoms-MS associated. Therefore, further studies will be necessary to evaluate the impact of different immune modulators on the cholinergic system activity both in the immune system and in the brain.

In conclusion, the data described in this review highlight as ACh and its receptors may be implicated in MS pathogenesis. However, further investigation on the genetic aspects and epigenetic modulation of cholinergic markers gene expression could significantly improve the knowledge about MS onset and progression as well as to contribute to identifying new possible therapeutic strategies.
